# Controllable Preparation of Ultrathin Sandwich-Like Membrane with Porous Organic Framework and Graphene Oxide for Molecular Filtration

**DOI:** 10.1038/srep14961

**Published:** 2015-10-12

**Authors:** Yuanzhi Zhu, Danyun Xu, Qingshan Zhao, Yang Li, Wenchao Peng, Guoliang Zhang, Fengbao Zhang, Xiaobin Fan

**Affiliations:** 1 School of Chemical Engineering and Technology, State Key Laboratory of Chemical Engineering, Collaborative Innovation Center of Chemical Science and Engineering, Tianjin University, Tianjin 300072, China

## Abstract

Porous organic frameworks (POFs) based membranes have potential applications in molecular filtration, despite the lack of a corresponding study. This study reports an interesting strategy to get processable POFs dispersion and a novel ultrathin sandwich-like membrane design. It was accidentally found that the hydrophobic N-rich Schiff based POFs agglomerates could react with lithium-ethylamine and formed stable dispersion in water. By successively filtrating the obtained POFs dispersion and graphene oxide (GO), we successfully prepared ultrathin sandwich-like hybrid membranes with layered structure, which showed significantly improved separation efficiency in molecular filtration of organic dyes. This study may provide a universal way to the preparation of processable POFs and their hybrid membranes with GO.

Combining adsorption and filtration is an efficient way to realize molecular separation for various applications with benefits of low energy consumption and easy operation^1^,^2^,^3^. Many well-known nanoporous materials such as zeolites and metal organic frameworks (MOFs) have been used in molecular separation processes^4^,^5^,^6^. As a nonmetallic counterpart of MOFs, porous organic frameworks (POFs) are formed by the covalent cross-linking of diverse organic moieties. Their pure organic nanostructure, tunable pore sizes and high permeability and stability endows POFs great promise for applications in molecular separation^7^,^8^, especially for water purification that is sensitive to metal contamination. However, corresponding studies have been hampered by its intrinsic hydrophobic property and agglomeration nature. As a consequence, the adsorptive separation using pristine POFs was usually conducted in the time and energy consuming batch condition, which is not suitable for practical applications. Therefore, it is desirable to assemble POFs into hydrophilic membranes for the fast and cost-effective molecular separation.

On the other hand, the separation mechanisms of the adsorptive membrane are mainly based on the interaction between the molecules and sorbents, including electrostatic adsorption, affinity adsorption and so on^9^. Despite the interaction can be affected by many factors (depending on the specific mechanisms involved), increasing the effective path length of the membrane is a common strategy to improve the separation efficiency^10^. For a same membrane, however, increase in the membrane’s thickness will result in an unavoidable increase in cost, an obvious decrease in the utilization of adsorption sites, as well as the great loss of flux and filtrate.

Inspired by the graphene oxide (GO) membranes^11^,^12^,^13^,^14^,^15^, we propose hereby an ultrathin sandwich-like membrane design (Fig. 1a), which can simultaneously increase the separation efficiency and the utilization of adsorption sites. Our proof-of-concept demonstration is based on the use of processable N-rich Schiff based POFs (SWN) (Fig. 1b) as the sorbent, and the GO nanosheets as two-dimensional (2D) interval layers. In this sandwich-like hybrid membrane, the horizontal microfluidic channels built by the GO can significantly increase the effective separation path in the POFs matrix without increasing their loading and thickness. Notably, during the molecular filtration, the hybrid membrane shows significantly better separation efficiency (Fig. 1c,d) and stability, which is highly desired in the pressure-driven molecular filtration process.

## Results

This study was started by an accidental finding during the interaction of SWN and lithium-ethylamine (Li-EDA). Our initial purpose was to isolate the SWN agglomerates by lithium intercalation and mutual electrostatic repulsion. This strategy has been successfully used to debundle carbon nanotubes^16^. When the SWN was added into the Li-EDA solution, we found that the blue color of Li-EDA gradually disappeared. This phenomenon should be attributed to the facile electron transfer^17^ from Li-EDA to SWN. At the same time, the SWN gradually swelled and formed homogeneous yellow dispersion. To our surprise, different from the treated carbon nanotubes that will immediately form antisolvent precipitation when dropped into water^18^, the obtained SWN could be readily dispersed and form processable dispersion (P-SWN) in water. On the other hand, pristine SWN could not be dispersed in water even after intensive sonication, because of its hydrophobic property and agglomerate structure.

**Figure 2:**
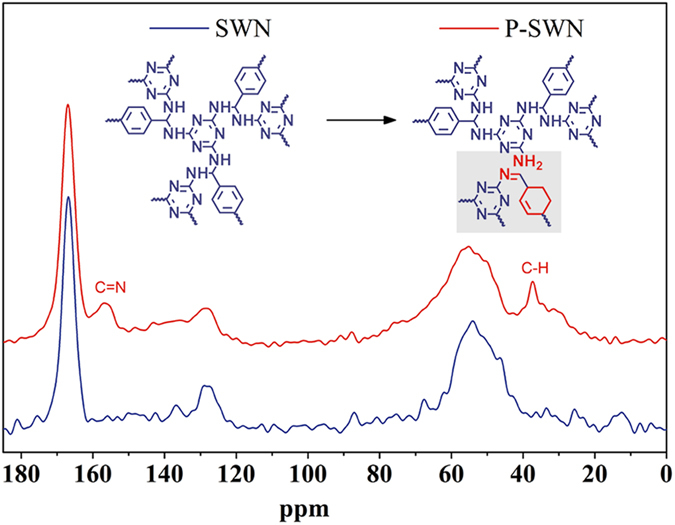
Solid-state ^13^C NMR spectra of pristine SWN (blue) and the obtained P-SWN (red). The two new resonance peaks at around 157 ppm and 38 ppm are signed to C =N and C−H bonds, respectively.

**Figure 3:**
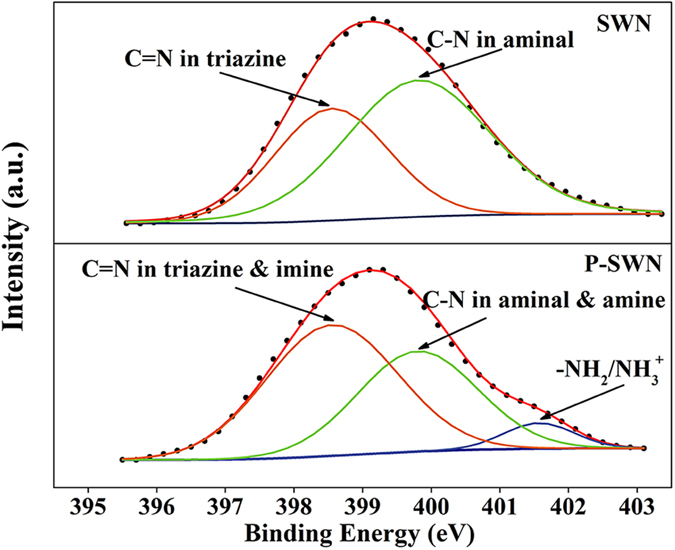
N 1s XPS spectra of pristine SWN and the obtained P-SWN. Note that the C−N decrease with the appearance of the protonated amine groups (−NH_2_/NH^3+^) after reacted with Li-EDA.

Subsequent careful characterizations revealed that SWN could undergo interesting reactions with Li-EDA. Compared with the pristine SWN, the ^13^C NMR spectra (Fig. 2) of the P-SWN shows two new resonance peaks at around 157 ppm and 38 ppm, which can be assigned to the C = N and C−H bonds, respectively^19^,^20^. In concert with the ^13^C NMR results, Fourier transform infrared spectroscopy (FTIR) reveals that the SWN has stronger stretching vibrations of C = N and C−H bonds after the treatment with Li-EDA (Fig. S1, Supporting Information). These results suggest the formation of C = N and aliphatic C−H bonds in the obtained P-SWN, probably caused by the deamination of the aminal linkages^21^ and the partial hydrogenation by Li-EDA. In brief, the aromatic rings in the SWN will be partially hydrogenated by Li-EDA, which is widely used for the hydrogenation of aromatic rings (Benkeser reaction)^22^,^23^,^24^. It should be noted that SWN was synthesized through the reaction of primary amines and the imine bonds in Schiff-base. Therefore, the formation of imine bonds should be attributed to corresponding reverse reactions, which may be caused by the strong basicity and reducing activity of Li-EDA. This hypothesis is confirmed by the X-ray photoelectron spectroscopy (XPS) (Fig. 3). According to previous studies^25^, the N 1s line of SWN can be deconvoluted into two main peaks with the binding energy of 398.55 eV and 399.8 eV, corresponding to the triazine units (C = N) and aminal linkages (C−N), respectively. After reacted with Li-EDA, the C−N decreases, while the C = N component (triazine and imine) increases, and the protonated amine groups (−NH_2_/NH_3_^+^) appear.

**Figure 4:**
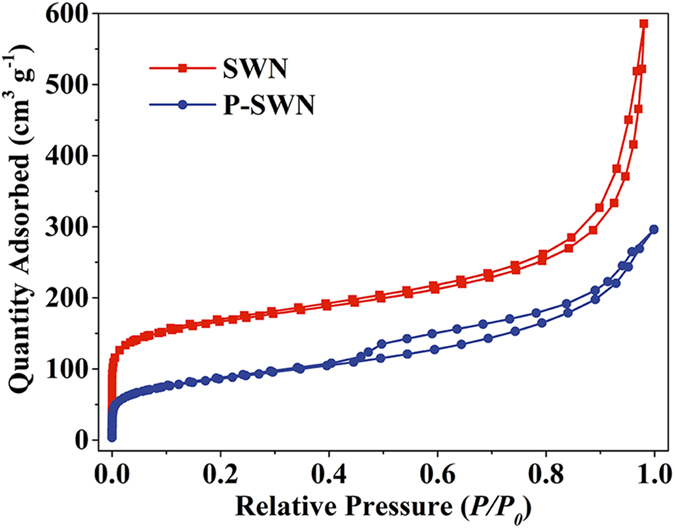
The N_2_ adsorption-desorption isotherms for SWN and P-SWN.

Generally, highly cross-linked structure and extended π-conjugation building block endow SWN and other POFs with a porous skeleton. This rigid structure and hydrophobic property makes them difficult to be dispersed in water or other solvents. We believe that the reduction of some aromatic rings and the formation of the hydrophilic imine and amine groups account for the swelling phenomenon and the improved dispersibility in water. A Direct evidence comes from the fact that the zeta potential of SWN increases from +9 mV to +36 mV (at pH = 7) after react with Li-EDA. Besides, the N_2_ adsorption-desorption isotherms (Fig. 4) reveal a decrease in adsorption capacity of steep gas uptake at relatively low pressure (*P/P*_*0*_ < 0.001), indicating the partly collapse of rigid aromatic skeleton during hydrogenation and deamination. The hysteresis loops were changed from H1-type to H4-type, which should be caused by the reduced agglomeration after the reaction.

Notably, despite the changes of pore structure (H−K and BJH analysis in Fig. S2) and the decrease in specific surface area (S_BET_) (decreased from 608 m^2^ g^−1^ to 299 m^2^ g^−1^), the saturated adsorption ability (*Q*_eq_) of P-SWG toward negatively charged MO increased significantly from 123 mg g^−1^ to 232 mg g^−1^ after the reaction (Table S1 in Supporting Information). On the contrary, the *Q*_eq_ toward positively charged MB remained a similar value. Based on these results, we can conclude that the adsorption ability of the adsorptive membrane is controlled by the interaction between the molecules and sorbent, in line with previous studies on molecular separation^26^,^27^. However, the change of the pore structure and porosity may also contribute to the mass transfer and flux during membrane filtration process.

**Figure 5:**
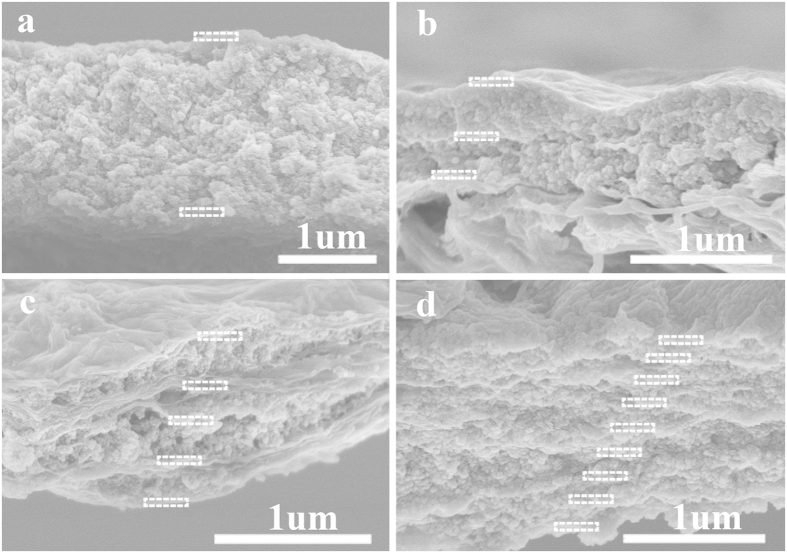
Cross-sectional SEM images for PSG-1 (a), PSG-2 (b), PSG-4 (c) and PSG-8 (d), respectively. The GO layers are highlighted with white rectangle. The total P-SWN loading is ~0.67 mg in every membrane, and the GO loading is ~3 mg m^−2^ in every GO layer.

To explore the potential of porous organic frameworks in molecular filtration, ultrathin sandwich-like membranes with intercalated layers of GO and P-SWN were prepared by the successive filtration of GO and P-SWN dispersion through a PTFE support. We proposed that the horizontal microfluidic channels built by the GO and POFs could significantly increase the effective path length and the stability of the membrane. Given the negative influence of GO on the water flux (Fig. S3, Supporting Information), GO loading of ~3 mg m^−2^ was chosen to deposit every intercalated GO layer, and the same amount of P-SWN was used in every membrane. The different layered structures can be easily controlled by varying the filtration processes. For example, SEM images of the obtained hybrid membranes with 1, 2, 4, 8 layers of P-SWN (named PSG-1, PSG-2, PSG-4 and PSG-8, P-SWN loading of ~0.67 mg) were shown in Fig. 5a–d, respectively. Despite the uneven surfaces, the thickness of the all the membranes are ~1 μm, as the same amount of P-SWN were used, and the thickness of GO can be ignored. It should be noted that the negatively charged GO and positively charged P-SWN layers can form a compact membrane because of their strong electrostatic attraction. In addition, the obvious corrugations on GO should contribute to a better water flux^28^,^29^.

**Figure 6:**
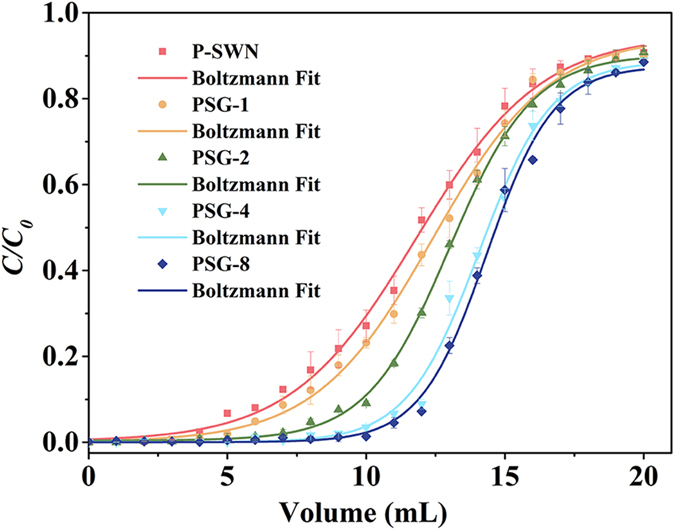
The experimental plots (the error bars represent the s.d. from three independent experiments) and Boltzmann fit of the breakthrough curves for the as-prepared hybrid membranes and P-SWN control. The feed concentration is 10 mg L^−1^ MO. The effective filtration area of the membrane is 4.1 cm^2^ with total P-SWN loading of ~0.67 mg. The applied pressure is fixed as 1 bar.

MO, a common organic contaminate in effluent, was used as the probe to study the filtration performances of these hybrid membranes. Normally, the as-prepared membranes with the PTFE support and P-SWN control were tested under an applied pressure of 1 bar. The breakthrough curves fit by Boltzmann model^30^ are summarized in Fig. 6. The breakthrough volume (*V*_*B*_) is the volume that corresponds to 1% of max concentration of the dye in the effluent, and the adsorption capacity (*Q*) corresponds to the dye amount adsorbed in membrane when effluent concentration reached 99% of the max concentration. Compared with the P-SWN control (pink line), the *V*_*B*_ of the membranes quickly increases with the increasing GO layer number until it reaches a stagnant when the GO layer number is over 4 (Table 1). The *Q* data show similar trend, which suggests the adsorption of the introduced GO can be neglected, attributed to its small loading and negatively charged nature^31^. Actually, the control experiment with a pure GO membrane (3 mg g^−1^) on the PTFE support demonstrates that limited MO could be retained. However, it should be noted that the introduction of GO will resulted in the decline of the flux (*ν*), especially when the P-SWN layer number is over 4.

Based on the Werkhoven-Goëwie model (described in Method)^30^,^32^, the theoretical plates (*N*)—the main index used to determine the effectiveness of a separation process—increase significantly when more GO layers are introduced in the sandwich-like membranes. The *N* of the PSG-4 membrane with a decent flux value can be calculated to be 21.83, which is about 4.5-fold of a pure P-SWN membrane. The improved separation efficiency could be explained by the increased effective path length and the higher utilization ratios of adsorption sites. In a pure P-SWN membrane, the flow will arbitrarily select the paths of less mass transfer resistance, resulting in the waste of abundant adsorption sites. Whereas the introduction of GO and the formation of horizontal fluidic channels can reduce the flow anisotropy and the flow death zones.

As expected, increasing the applied pressure could increase the flux but resulted in the degradation of the separation efficiency (Fig. S4 and insert). When the applied pressure reaches 3 bar, the flux of PSG-4 increases to 4181 ± 721 L m^−2^ h^−1^, and the Q decreases to 100.7 μg. In comparison, control experiments with a pure P-SWN membrane show the concentration of the effluent quickly reaches the feed concentration. These results indicate that the sandwich-like hybrid membrane has higher stability than the membrane without GO, especially for the filtration process under a higher pressure.

**Figure 7:**
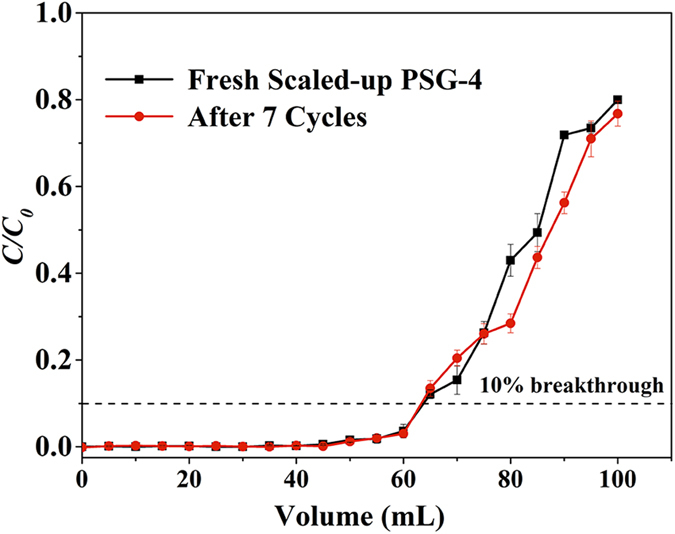
Cycling test of the scaled-up PSG-4 membrane. The breakthrough curves for fresh scaled-up PSG-4 (black line) and the membrane after 7 cycles (red line). The error bars represent the s.d. from three independent experiments. The feed concentration is 20 mg L^−1^ MO. The effective filtration area of the membrane is 4.1 cm^2^ with P-SWN loading of ~6.7 mg. The applied pressure is fixed as 1 bar.

It should be noted that the breakthrough volume and adsorption capacity of the membranes can be easily scaled up by increasing the loading of P-SWN. Despite the increased feed concentration (20 mg L^−1^), the *V*_*B*_ of a scaled-up PSG-4 membrane with P-SWN loading of ~6.7 mg can reach 47 ml, which means an MO adsorption capacity of 140.3 mg g^−1^ (Fig. 7). Importantly, the filtrated volume of the scaled-up PSG-4 at 10% breakthrough keeps constant even after 7 cycles (Fig. 7 and Fig. S5), while that of the P-SWN counterpart reduced gradually from 56 mL to 18 mL, probably due to the disassembly of P-SWN from the PTFE support.

In summary, we found that the N-rich Schiff based POF (SWN) could react with Li-EDA and formed processable dispersion (P-SWN). It is believed that the reduction of many aromatic rings and the formation of the new hydrophilic imine and amine groups account for the excellent dispersibility of P-SWN in water. We also demonstrated that the successively filtrating the obtained P-SWN and GO could be used to prepare a unique sandwich-like membrane with significantly improved separation efficiency in molecular filtration. This novel membrane structure with horizontal fluidic channels can significantly increase the effective path length and increases the utilization of adsorption sites. The strategy and methods used here may be readily changed to prepare other processable POFs and hybrid membranes for applications in molecular filtration.

## Methods

### Synthesis of SWN-1

Melamine (313 mg, 2.485 mmol), para-phthaladehyde (500 mg, 3.728 mmol) and dimethyl sulfoxide (15.5 ml) were added to a dried Schlenk flask fitted with a condenser and a magnetic stirring bar. After being degassed with argon bubbles, the mixture was heated to 453 K for 72 h under an inert atmosphere. When reaction completed and the reactor cooled down to room temperature, the precipitated SWN-1 powder was isolated via filtration over a Büchner funnel and washed with excess tetrahydrofurane and dichloromethate. The solvent was removed under vacuum at room temperature to afford the materials as off-white powders in 55% yield.

### Preparation of P-SWN aqueous dispersion

The lithium amide solution (Li-EDA) was prepared as follows. Firstly, 100 mL of dry ethylenediamine (EDA) was added to an argon-purged Schlenk flask. Then, 100 mg of Li was added at room temperature. Dissolution of Li was fast with the formation of a blue solution (solvated electron). The as-prepared SWN-1 (100 mg) was added to this solution, and the solution was stirred at room temperatures for 1 h to get uniform yellow SWN-1 dispersion. The resulting dispersion was dropped into water to obtain P-SWN aqueous dispersion.

### Preparation of GO aqueous dispersion

The GO aqueous dispersion was prepared by adding a certain amount of GO (prepared by Hummer’s method) into water followed by 1 h ultrasonication. The resulting dispersion was centrifuged at 10000 rpm for 30 min and the supernatant was collected.

### Fabrication of sandwich-like membrane

P-SWN and GO dispersions were alternately filtrated through PTFE support with 0.22 μm pore size. To ensure consistency between membranes with different GO layer, the P-SWN loading was kept as a constant. Once the filtration was completed, 10 ml of HCL (0.1 M) were filtrated to remove the remaining ethylene diamine and Li ions. The resultant membrane was washed by deionized water until the effluent is neutral. All membranes were stored in water to keep wet before use. The film is uniform and coherent but does not, in this first example, have sufficient mechanical strength to be self-supporting upon removal.

### Membrane filtration and cycling experiments

The filtration experiments were conducted with a dead-end stirred cell (Model 8010, Millpore Co., USA. The volume capacity is 10 mL and the effective area of the membrane is 4.1 cm^–2^). In this study, all membranes were tested without removing the PTFE support. The as-prepared membrane was placed in the cell and dye solutions with different initial concentration were filtrated through the membrane at desired pressure. The effluent at each stage was collected and analyzed. In the membrane recycle stage, 30 mL of eluent (1 M HCL in 80% methanol) was filtrated through the membrane. Then the membrane was washed by filtrating deionized water to neutral for another cycle.

### Breakthrough curves fit and calculation of breakthrough volume (*V*_*B*_) and theoretical plate (*N*)

The experimental plot of breakthrough curves can be fitted by Boltzmann model as Eqs. (1):


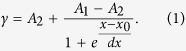


Where *γ* represents the ration of the effluent to the feed concentration, *x* is the volume of effluent, *A*_*1*_ and *A*_*2*_ are two regression parameters.

Retention volume (*V*_*R*_) and Breakthrough volume (*V*_*B*_) were calculated as expressed in Eqs. (2) and (3), respectively:


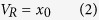



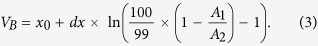


The theoretical plate can be calculated by Werkhoven-Goëwie model as Eqs. (4):


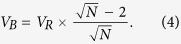


## Additional Information

**How to cite this article**: Zhu, Y. *et al.* Controllable Preparation of Ultrathin Sandwich-Like Membrane with Porous Organic Framework and Graphene Oxide for Molecular Filtration. *Sci. Rep.* **5**, 14961; doi: 10.1038/srep14961 (2015).

## Supplementary Material

Supplementary Information

## Figures and Tables

**Figure 1:**
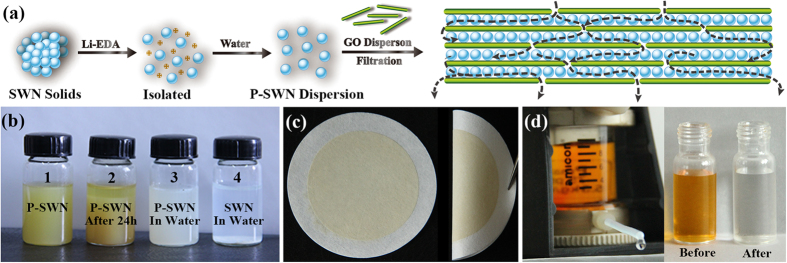
(**a**) Illustration for the preparation of the sandwich-like membrane. (**b**) Digital photo of P-SWN and pristine SWN dispersion with concentration of ~0.5 mg mL^−1^. (**c**) Digital photo of a hybrid membrane on PTFE support. (**d**) Digital photos of filtration equipment and the MO solutions (10 mg L^−1^) before and after filtration.

**Table 1:** The breakthrough parameters from Fig. 6 and corresponding water flux.

	*V*_*B*_ (mL)	*Q* (μg)	*N*	*ν* (L m^–2^ h^–1^)
P-SWN	1.02	121.66	4.79	4027.75 ± 184
PSG-1	2.56	127.46	6.36	2832.42 ± 230
PSG-2	5.24	136.24	11.22	2310.65 ± 110
PSG-4	8.05	147.20	21.83	1951.22 ± 98
PSG-8	8.80	150.32	26.82	934.09 ± 68

*V*_*B*_, the volume that corresponds to 1% of max concentration of the dye in the effluent; *Q*, dye amount adsorbed in membrane when effluent concentration reached 99% of max concentration; *N*, theoretical plates; *ν*, water flux.
