# Supplementary material for: Controllable Preparation of Ultrathin Sandwich-Like Membrane with Porous Organic Framework and Graphene Oxide for Molecular Filtration
Source: Sci Rep. 2015 Oct 12;5:14961. doi: 10.1038/srep14961 (PMC4601088; doi:10.1038/srep14961)
Supplement: Supplementary Information [file srep14961-s1.pdf]

Controllable Preparation of Ultrathin Sandwich-Like Membrane with Porous Organic Framework and Graphene Oxide for Molecular Filtration

Yuanzhi Zhu, Danyun Xu, Qingshan Zhao, Yang Li, Wenchao Peng, Guoliang Zhang, Fengbao Zhang and Xiaobin Fan*

School of Chemical Engineering and Technology, State Key Laboratory of Chemical Engineering, Collaborative Innovation Center of Chemical Science and Engineering, Tianjin University, Tianjin 300072, China. E-mail: xiaobinfan@tju.edu.cn Fax: +86-22-27890090

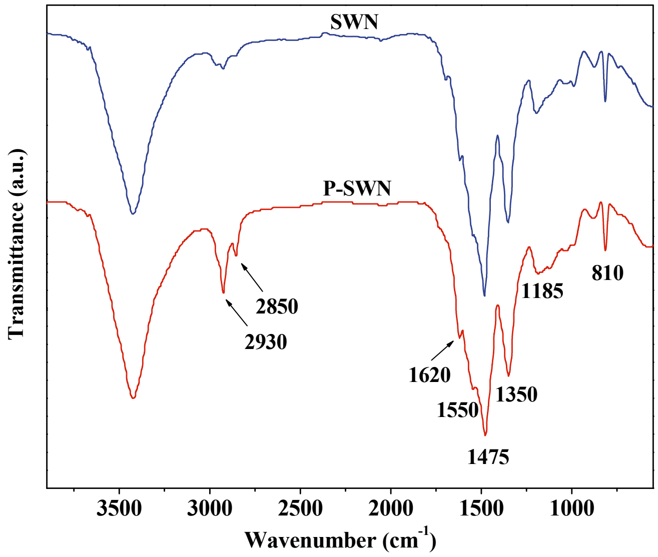

Figure S1 | Fourier transform infrared (FTIR) spectra of SWN and P-SNW.

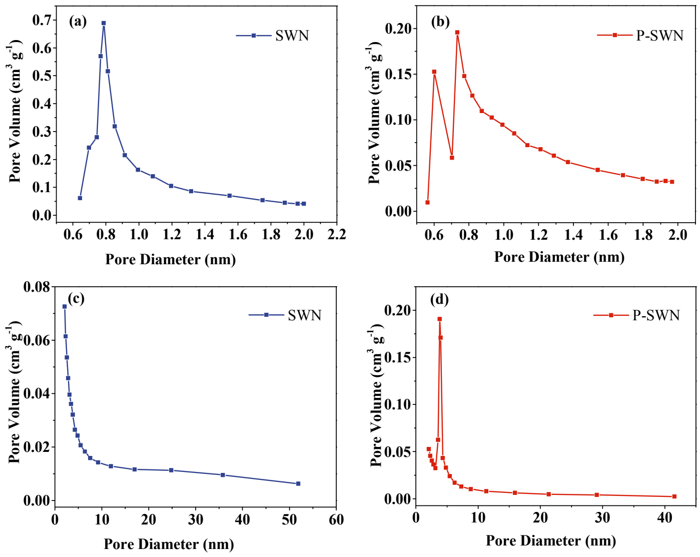

Figure S2 | The pore sizes distribution of SWN and P-SWN. Microporous size distributions are estimated by Horvath-Kawazoe (HK) methods in (a) and (b). Mesoporous size distributions are estimated by Barrett-Joyner-Halenda (BJH) methods in (c) and (d).

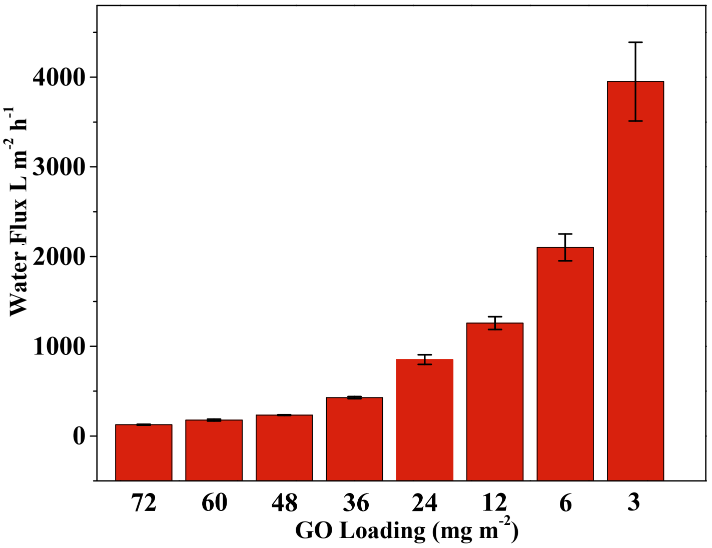

Figure S3 | The water permeance of GO membranes with various loading. The applied pressure is fixed as 1 bar.

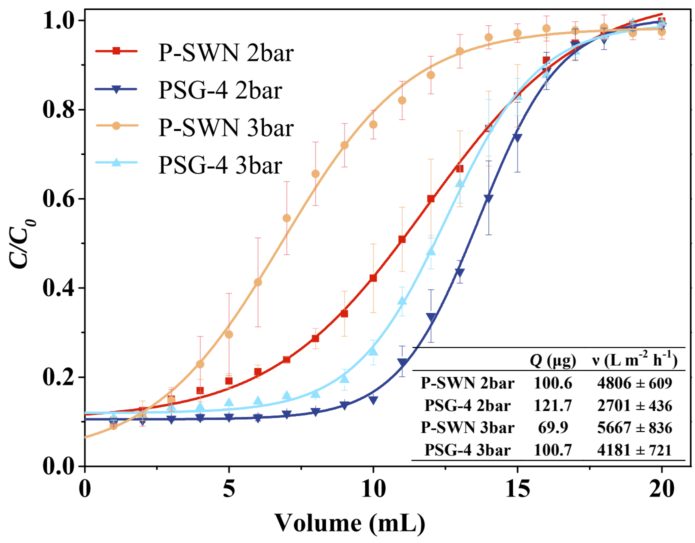

Figure S4 | The experimental plots (the error bars represent the s.d. from three independent experiments) and Boltzmann fit of the breakthrough curves for the as-prepared hybrid membranes and P-SWN control. The feed concentration is 10 mg L^{-1} MO. The effective filtration area of the PSG membrane is 4.1 cm^2 with total P-SWN loading of ~0.67 mg. The applied pressure is fixed as 2 bar or 3 bar. Insert: The dye amount adsorbed on the membrane (Q) and the water flux (v).

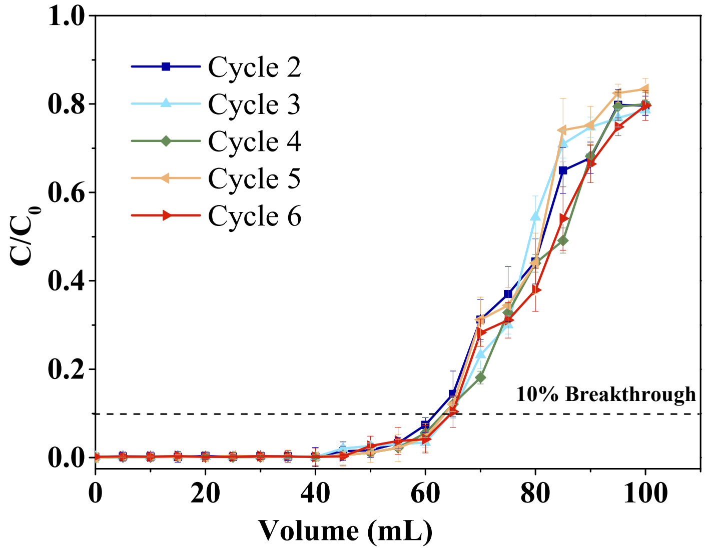

Figure S5 | Recycling runs of the sandwich-like membrane corresponding to Figure 4 in the manuscript. The feed concentration is 20 mg L^{-1} MO. The effective filtration area of the PSG membrane is 4.1 cm^2 with P-SWN loading of ~6.7 mg. The applied pressure is fixed as 1 bar.

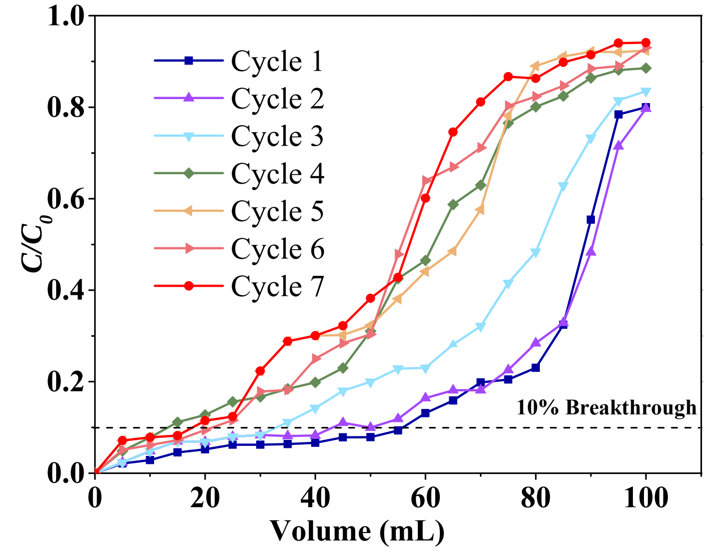

Figure S6 | Recycling runs of a thicker P-SWN membrane. The feed concentration is 20 mg L^{-1} MO. The effective filtration area of the PSG membrane is 4.1 cm^2 with P-SWN loading of ~6.7 mg. The applied pressure is fixed as 1 bar.

Table S1. Saturated adsorption capacities of SWN and P-SWN for methyl orange (MO) and methylene blue (MB).

Absorbent Samples	Q_{eq} (mg g^{-1})	
	MO (positive charged)	MB (negative charged)
SWN	123	116
P-SWN	232	121
